# Advances and challenges in management of large vessel vasculitis

**DOI:** 10.2478/rir-2023-0028

**Published:** 2023-12-19

**Authors:** Cong-Qiu Chu

**Affiliations:** Division of Arthritis and Rheumatic Diseases, Oregon Health & Science University, Portland Oregon 97239 USA; Rheumatology Section, Veterans Affairs Portland Health Care System, Portland Oregon 97239 USA

**Keywords:** large vessel vasculitis, giant cell arteritis, takayasu arteritis, glucocorticosteroids, disease modifying antirheumatic drugs

## Abstract

Glucocorticoids (GC) remains the mainstay for management of large vessel vasculitis (LVV). Recent introduction of interleukin-6 signaling blocker, tocilizumab has substantially changed the practice in management of patients with LVV, in particular, giant cell arteritis (GCA). Benefit of tocilizumab to patients with Takayasu arteritis (TAK) is supported by observational studies, but randomized clinical trials are lacking. Addition of tocilizumab enables reduction of the total amount of GC in patients with GCA, but GC burden remains high and to be further reduced. Ongoing studies aim at minimal use of GC or even GC-free. Tumor necrosis factor inhibitors appear to be beneficial to TAK despite their ineffectiveness to GCA. Randomized clinical trials are undergoing to target other inflammatory cytokines in both GCA and TAK. Janus kinase inhibitors alone or in combination with conventional disease modifying anti-rheumatic drugs showed promising results in treatment of TAK.

## Introduction

Management of large vessel vasculitis (LVV) can be challenging. The introduction of interleukin (IL)-6 receptor blockade has significantly changed our approach to the management of LVV, particularly giant cell arteritis (GCA), but glucocorticoids (GC) remains the mainstay for medical treatment of LVV.^[1]^ Research effort has focused on reducing the burden of GC by testing different regimens of GC-sparing agents including IL-6 receptor targeted therapy, combination of conventional disease modifying anti-rheumatic drugs (DMARDs), other biological DMARDs, and JAK inhibitors (JAKi). For Takayasu arteritis (TAK), mostly based on extrapolated information from GCA, several recent clinical studies for treating TAK with new regimens or biological DMARDs have been conducted and some have shown promising results.

It was once thought that GCA and TAK represent conditions in the same spectrum since both are characterized by granulomatous inflammation involving the entire arterial wall,^[2]^ but there are distinctive differences between GCA and TAK despite similarities in histopathological changes. GCA occurs in adults of 50 years and older while TAK most commonly affects individuals of 40 years of age or younger.^[3]^ The age cut-off as one of the criteria in the American College of Rheumatology (ACR) classification of the two conditions appears to be arbitrary but may actually reside in the different physiological status of host immune system and the vascular biology in the two age groups.^[4, 5, 6]^ The difference between GCA and TAK is also reflected in response to therapeutic agents. For example, tumor necrosis factor inhibitors (TNFi) had no benefit for GCA, but appeared to be efficacious for TAK. Thereby, developing novel targeted therapies for LVV relies on advancement in understanding the pathogenesis and also in recognition of the difference between GCA and TAK.

## Advancement in Management of GCA

IL-6 is a pleiotropic inflammatory cytokine and is a major cy-tokine to mediate acute phase protein release. Dysregulation of IL-6 plays a critical role in the onset and development of several chronic inflammatory conditions including GCA. Elevated serum levels of IL-6 are detected in majority of untreated GCA patients and correlate with disease activity.^[7,8]^ It is likely that IL-6 is produced in the lesional artery since increased levels of mRNA for IL-6 are detected in situ in GCA temporal biopsy samples.^[9]^ Tocilizumab, a monoclonal antibody to IL-6R, blocks IL-6 signaling and was initially approved for treating rheumatoid arthritis (RA). The landmark study, GiACTA trial led to the approval of tocilizumab for treatment of GCA by US Food and Drug Administration (FDA) and European Medicines Agency (EMA).^[1]^ The addition of tocilizumab to regimen of GC based therapy substantially reduced the burden of GC in GCA patients.^[1]^ Both ACR and European Alliance of Associations for Rheumatology (EULAR) guidelines recommend use of tocilizumab in management of GCA.^[10,11]^ The extended open-labeled study of GiACTA showed no new safety signal in 3 years and confirmed that tocilizumab weekly dosing is superior over biweekly dosing in both newly diagnosed and relapsing GCA at their initial entry of the study.^[12,13]^ Interestingly, in the extended period of observation, 42% who achieved remission on tocilizumab weekly at the end of year 1 has remained in GC-free remission for two years without continuation of tocilizumab; relapse in some patients can be managed by tocilizumab alone without addition of GC.^[13]^ These data suggest that in practice tocilizumab will need to be prescribed as weekly dosing, and tocilizumab can be stopped after treatment for one year in some patients. Besides, in some, especially in relapse population, tocilizumab alone may be sufficient to achieve remission but this needs to be prudent since there is no evidence to indicate that tocilizumab alone is sufficient to manage vision impairment.^[13]^

However, several issues associated with tocilizumab in treatment of GCA remain. First, ultimately, over half of GCA patients in GiACTA trial treated with tocilizumab still flared over three years, yet had long-term remission.^[1,12,13]^ Naturally, identifying these two subgroups is required to guide clinical practice, but this effort has not yet been successful.^[14]^ Second, tocilizumab is a potent suppressor of acute phase reactants and can rapidly normalize C-creative protein (CRP) and erythrocyte sedimentation rate (ESR) but not necessarily suppress the underlying inflammation of vasculitis. Since CRP and ESR are integral components of GCA remission and relapse criteria, tocilizumab induced normalization of CRP and ESR may falsely impress both patients and physicians about actual status of GCA. Indeed, only one-third of GCA patients showed normalized signal in arterial wall on magnetic resonance angiography (MRA) or ^18^F-fluorodeoxyglucose (^18^F-FDG) uptake in positron emission tomography (PET/CT) scans even they were in clinical and laboratory remission after being treated with tocilizumab for 52 weeks.^[15,16]^ Ideal biomarkers which are not directly affected by tocilizumab are required. MRA or PET/CT may serve such a role, but cost and inconvenience make either unsuitable for practice. Third, whether tocilizumab is effective on large-vessel-GCA (LV-GCA) remains unknown. LV-GCA is associated with more flares and requires higher amounts of GC.^[17]^ Fourth, use of tocilizumab in real world practice. Several retrospective studies confirmed efficacy of tocilizumab in treating GCA ^[18, 19, 20, 21, 22, 23]^ as demonstrated by rapid reduction of symptoms, vision benefit and reduction of polymyalgia rheumatica (PMR) symptoms, but with an increased rate of infections, no effect on severe outcomes such as cardiovascular complications.^[19,21]^ The prescription of tocilizumab in real world practice is primarily for relapsed and refractory, GC-dependent GCA patients and is considered an under-utilization ^[22]^ although ACR guidelines endorsed use of tocilizumab to all GCA patients, newly diagnosed and relapsed.^[11]^ Practicing physicians are also reluctant to prescribe tocilizumab to GCA patients who have history of diverticular disease which affect over half of the population of aged > 50 years.^[24]^ It is less clear how long patients should remain on tocilizumab. Gradually tapering off tocilizumab after remission is achieved and maintained for one year seemed a reasonable approach.^[25]^ Yet high quality studies with a substantial amount of data will be required to collect from newly diagnosed GCA patients in real world practice to determine the duration of tocilizumab treatment.

Despite the therapeutic efficacy of tocilizumab, GC is still not completely spared from the treatment regimen of GCA. Because of its proven rapid onset of anti-inflammatory effect, high dose of GC is highly recommended for prompt initiation in newly diagnosed GCA to prevent the potential sever ischemic complications of GCA.^[10,11]^ In practice, high dose of GC is actually started if the diagnosis of GCA is suspected before its confirmation by temporal artery biopsy or imaging studies. The recommended initial dose of oral GC, commonly prednisone, is different. That is, 40–60 mg/day per EULAR versus 1 mg/kg/day (up to 80 mg/day) per ACR guidelines.^[10,11]^ The initial dose will affect the subsequent tapering regimen and cumulative dose of GC. There is no high quality evidence comparing GC tapering schedules to guide practice.^[26]^ The cumulative amount of GC is directly associated with an increased risk of serious GC-related adverse effects ^[27, 28, 29]^ which result in increased health care costs.^[30]^ Improving benefit-risk ratio to mitigate GC-related adverse effects continues to be a challenge.^[31]^

Biological DMARDs for other targets are being actively studied in GCA. Since both T helper type 1 (Th1) and Th17 cells are implicated in immune mechanisms of GCA,^[32]^ abatacept that inhibits T cell activation has been tried for GCA (NCT00556439).^[33]^ In this phase II clinical trial, addition of intravenously administered abatacept (10 mg/kg every 4 weeks after loading doses) was shown to be superior than GC alone in maintaining clinical remission in newly diagnosed or relapsed GCA.^[33]^ In an ongoing phase III study (ABAGART; NCT04474847), subcutaneously administered abatacept (125 mg weekly) is also being tested whether in combination with GC is superior in induction and maintenance of clinical remission for patients with GCA. Neither of these studies tested whether abatacept will have the capacity to reduce the burden of GC.

Interleukin-23 (IL-23)/Th17 pathway specific targeted therapies are all being investigated in the setting of fast GC reduction regimen, i. e., 26-week tapering off in newly diagnosed and relapsed GCA patients. These includes phase II (NCT03765788)^[34]^ and III (NCT04930094) trials with secukinumab (anti-IL-17A), phase II with guselkumab (anti-IL-23p19; NCT04633447) and ustekinumab (anti-IL-12/23p40; NCT03711448). Scientific rationale underlies these studies is well justified, but the clinical efficacy is to be determined, in particular, whether these biological DMARDs are able to spare GC. Recently published phase II, proof-of-concept (NCT03765788) trial with secukinumab reported promising results in patients with newly diagnosed or relapsing GCA.^[35]^ In this study, corticosteroids were tapered off at week 26. Seculinumab (*n* = 27; loading does at 300 mg/week for 4 weeks, then 300 mg every 4 weeks) induced remission in 70% of GCA patients while only 20% in remission in placebo treated group (*n* = 25) at week 28. However, the sustained therapeutic effect of secukinumab in GCA is yet to be confirmed by phase III studies.

Granulocyte-macrophage colony stimulating factor (GM-CSF) has implicated in the pathogenesis of GCA.^[36,37]^ In the wall of GCA temporal artery biopsy samples infiltrating macrophages expressing CD206 and matrix metteloproteinase-9 (MMP-9) are associated with tissue destruction. GM-CSF highly regulates CD206 expression by macrophages and GM-CSF is in close vicinity with these CD206^+^/MMP-9^+^ macrophages suggesting that GM-CSF may drive the tissue destructive phenotype macrophages in GCA.^[36]^ A monoclonal antibody to GM-CSF receptor-α, mavrilimumab in a proof-of-concept study (NCT03827018) was tested for its efficacy and GC sparing capacity in 26-week tapering of prednisone. Mavrilimumab treated group achieved clinical remission at 26 weeks in 83% GCA patients versus 50% in placebo treated group.^[38]^ This promising result requires further validation in a larger group of patients and longer-term observation.

JAKi are small molecules which are highly efficacious in treatment of inflammatory diseases such as RA for their potent blocking effect on signaling transduction of multiple cytokines. JAKi is effective in suppression of immune response in human-mouse xenograft model of LVV.^[39]^ Case series showed that JAKi in combination with GC were able to induce and maintain clinical remission of GCA.^[40, 41, 42]^ In a pilot proof-of-concept study, 15 patients with relapsing GCA were treated with baricitinib; 13/15 patient achieved GC-free remission in 52 weeks observation.^[43]^ Upadacitinib, a JAK1 selective inhibitor is being tested in a phase III trial (NCT03725202) for treating GCA in a 26-week GC tapering regimen.

Sparing GC and maintaining remission has always been the goal for management of GCA. Both EULAR and ACR recommend methotrexate if tocilizumab is contraindicated or not suitable ^[10,11]^ since the evidence for methotrexate as adjuvant therapy for GCA is not as strong as for tocilizumab.^[44, 45, 46, 47, 48]^ It is noteworthy that the dose of methotrexate used in these studies is rather lower (7.5 – 15 mg/week) than that used in other vasculitides. It is to be determined if a higher dose of methotrexate would be more effective for GCA.

Encouraging results come from a pilot study aiming at minimum of GC dosing. GCA patients were treated with intravenously administration of methylprednisolone 500 mg/day for 3 consecutive days, followed by tocilizumab monotherapy without oral GC; 78% (14 out of the 18 GCA patients) achieved remission within 24 weeks and 72% remained remission at week 52.^[49]^ This treatment regimen must be confirmed by long-term studies with a larger number of patients in comparison with standard of care. It would be interesting to explore whether combination therapy of tocilizumab with methotrexate would be superior than either tocilizumab or methotrexate monotherapy after discontinuation of the ultra-short course of GC. As it has been demonstrated that in management of RA combination of methotrexate with a biological DMARD is safe and well-tolerated, and efficacy is superior than a biological DMARD monotherapy. Future studies should aim at total elimination of GC after the initial bridging treatment. This goal may be achieved by combination of methotrexate with a biological DMARD or JAKi.

## Advancement in Management of TAK

Like that for GCA, high dose of GC remains the mainstay for induction of remission for TAK management. Moreover, low dose of GC, e. g. prednisone up to 10 mg daily is often used for maintenance of remission ^[50];^ even so, more than 50% of patients will experience relapse.^[51]^ For this reason, both EULAR and ACR recommend DMARDs to be used in combination with high dose of GC at the initiation of treatment.^[10,11]^ Choices of DMARDs included methotrexate, azathioprine, mycophenolate, leflunomide, and cyclophosphamide, but the strength of evidence for the efficacy of these DMARDs is rather low since they all derived from cohort studies or retrospective observations and with a small number of patients rather than randomized clinical trials (RCT).^[52, 53, 54, 55]^ It is widely recognized that it is difficult to conduct an RCT with a large number of patients due to the low prevalence of TAK.

However, two recent cohort based prospective studies using East China Takayasu Arteritis (ECTA) cohort of patients yielded interesting results.^[56,57]^ Leflunomide has comparable efficacy to that of tofacitinib in treatment-naïve and relapse TAK patients,^[56]^ but tofacitinib is superior to methotrexate in induction of clinical remission and prevention of disease relapse.^[57]^ For further confirming the findings in efficacy of methotrexate versus tofacitinib, a phase IV RCT (NCT04299971) is being conducted. In addition, a phase III RCT is ongoing to explore the efficacy of upadacitinib (NCT04161898) along with prednisone.

TNF is associated with granuloma formation which is seen in both GCA and TAK arteries. Serum TNF levels correlate with disease activity in TAK.^[58]^ Unlike that in GCA, TNFi appeared to be effective in treating TAK according to observational studies.^[26]^ A recent retrospective multicentered observational study showed TNFi has a similar efficacy to that of tocilizumab in refractory TAK patients.^[59]^ Interestingly, TAK patients appear to be able to tolerate higher doses of infliximab. For example, young refractory TAK patients were treated with up to 12 mg/kg of infliximab every 4 weeks without increased risk of infections.^[60]^ The higher than usual dose of TNFi should be further tested in a larger number of patients and for long-term observation for efficacy and safety. TNFi in combination with methotrexate in treating RA patients has been a common practice and well tolerated by most RA patients, a trial of such a combination should be explored in TAK patients.

In addition to case reports on effectiveness of tocilizumab for refractory TAK, an observational study showed that tocilizumab (subcutaneous injection at 162 mg/week) in combination with GC was superior to GC alone or GC in combination with methotrexate or azathioprine for relapse-free survival at 24 weeks.^[61]^ In another observational study, 57 treatment-refractory TAK patients were treated with tocilizumab (most with intravenous infusion), 75% patients achieved clinical remission at 12 months, but only 21% showed complete improvement on imaging test.^[62]^ In treatment-naïve TAK patients, tocilizum-ab was tested whether GC can be discontinued earlier than the duration of the standard care. Tocilizumab was given at 8 mg/kg, intravenously infusion, monthly for 6 months along with GC, 85% TAK patients achieved remission at 6 months, and 54% patients was able to discontinue GC. However, in extended observation period of 12 months, 54% patients had relapse.^[63]^ These results suggest that tocilizumab may be used as GC sparing agent, but long-term treatment is required to maintain remission. In an RCT, tocilizumab (subcutaneous injection at 162 mg/week) in combination with GC was tested for treating relapse TAK patients, the primary endpoint, time to relapse was not met,^[64]^ but tocilizumab was shown to be able to spare GC in long-term extension study at 96 weeks.^[65]^ These findings suggest that tocilizumab is able to spare GC in long-term in relapsed TAK patients. Importantly, imaging assessment showed arteritis in most of patients are stable or improved.^[65]^ Nonetheless, tocilizumab has been recommended by EULAR and ACR as second line agent in TAK patients who are resistant to other DMARDs.^[10,11]^

Based on recent findings from studies on pathogenesis of TAK, biological DMARDs targeting T cells, cytokines of Th17 pathway of Th17 pathway and B cells including abatacept, ustekinumab, secukinumab, and rituximab have all been explored for treating TAK.

Like in GCA, Th17 and Th1 cells may be involved in the pathogenesis of TAK; Th17 and Th1 related cytokines were highly elevated in the peripheral blood of TAK patients and the levels of these cytokines correlated with disease activity. Moreover, interferon (IFN)-γ and IL-17A present at the lesions of vasculitic arteries of TAK.^[66]^ In addition, an increased number of tissue infiltrated CD8^+^ T cells were found in TAK than in GCA although their exact effect yet to be elucidated.^[67,68]^ Despite the implication of T cells in mediating the disease process in TAK, an RCT for abatacept failed to show efficacy as GC sparing agent for TAK.^[69]^ However, therapies targeting Th17 pathway have reported promising results. For instance, Refractory cases of TAK treated with ustekinumab reported positive results^[70,71];^ an RCT for ustekinumab with an intravenous loading dose is being conducted for treating relapsing TAK (NCT04882072), aiming to taper off GC at week 52. Most recently, Tian *et al* ^[72]^ reported that in a prospective open-labeled clinical trial, secukinumab (anti-IL-17A) showed as effective as TNFi for treating TAK who were refractory to GC in combination with conventional DMARDs or tocilizumab. At 3 months, secukinumab-treated (*n* = 19) TAK patients achieved a 31.8% response rate (complete and partial response combined) while TNFi-treated (*n* = 34) response was 58.8% (*P* = 0.057); and at 6 months, the response rate was 52.6% and 64.7% in secukinumab and TNFi (*P* = 0.39) respectively. These results suggest that IL-17A (and even IL-23) might be a valid target for TAK although RCT studies with a larger number of patients will be required to affirm the efficacy of IL-17A blockade.

The role of B cells and autoantibodies in the pathogenesis of TAK was not well delineated until recently although autoantibodies against aorta were described in TAK patients previously. B cells are found in the adventitia of TAK artery and appear to be in a larger number compared with that in GCA samples, implying their involvement in the disease process in TAK.^[67]^ Moreover, the number of CD19^+^/CD20^-^/CD127^high^ antibody secreting B cells is significantly higher in TAK patients with active disease.^[73]^ Mutoh *et al*.^[74]^ identified two autoantibodies in Japanese TAK patients: anti-endothelial protein C receptor (EPCR) and anti-scavenger class B type I (SR-BI) antibodies; several features of these two autoantibodies indicate that they are likely to be pathogenic for TAK. Interestingly, not all TAK patients produce these auto-antibodies; anti-EPCR is detectable in 34.6% while anti-SR-BI in 36.5% TAK patients. Thus, TAK patients can be stratified into three clinical phenotypes based on the presence of these autoantibodies, *i.e*., anti-EPCR positive, anti-SR-BI positive and double antibody negative. Using human protein array and unbiased screening assay, Wen *et al* ^[75]^ identified 8 novel specific autoantibodies in patients with TAK. Thereby, profiling these autoantibodies may have implications in stratifying patients for B cell targeted therapy, such as rituximab. Case reports of rituximab on TAK reported contradictory results and no RCT is being conducted.^[76]^ In retrospective case series studies, rituximab, a chimeric therapeutic antibody depleting CD20^+^ B cells has been shown to be effective in some treatment-refractory TAK patients.^[73,77]^ RCTs are needed to verify the efficacy of rituximab for TAK in a larger number of patients who are preferably stratified based on presence of autoantibodies. It would be interesting to expect whether TAK patients with autoantibodies are responsive to rituximab treatment.

## Concluding Remarks

GCA and TAK are LVV affecting two distinct populations of older and younger adults respectively, and TAK can affect children as well. Current management of LVV is GC based. Research effort has been devoted to sparing GC with im-munosuppressants and advancement has been made with using biological DMARDs and JAKi for reduction of overall GC burden, but GC-free remission is still rare. Clinical studies will need to focus on testing regimens with currently available DMARDs aiming at elimination of GC for long-term remission. Combination of a biological with a conventional DMARDs might be an option to achieve this goal.
